# Effectiveness of Spices and Herbs in Improving Diet Quality Among Underserved Populations

**DOI:** 10.1093/nutrit/nuag012

**Published:** 2026-05-26

**Authors:** Christopher R D’Adamo, Patrick F McArdle, Gabriela Piedrahita, Mark K Bahr-Robertson, Brian M Berman, Jonathan M Scott

**Affiliations:** Department of Family & Community Medicine, University of Maryland School of Medicine, Baltimore, MD 21201, United States; OvationLab, Richmond, VA 23220, United States; Department of Medicine, University of Maryland School of Medicine, Baltimore, MD 21201, United States; OvationLab, Richmond, VA 23220, United States; Nova Institute for Health, Baltimore, MD 21231, United States; Department of Family & Community Medicine, University of Maryland School of Medicine, Baltimore, MD 21201, United States; Nova Institute for Health, Baltimore, MD 21231, United States; Department of Military and Emergency Medicine, Consortium for Health and Military Performance, Uniformed Services University, F. Edward Hébert School of Medicine, Bethesda, MD 20814, United States

**Keywords:** spices, herbs, diet quality, MyPlate, underserved, National School Lunch Program, military

## Abstract

**Background:**

Diet quality in the United States is lacking and in need of improvement. Many populations with financial constraints, limitations to accessing healthy foods, and other social determinants of health face additional barriers to eating a healthy diet. The sensory-enhancing properties of affordable and widely accessible spices and herbs were hypothesized to be a promising vehicle in helping surmount some of the barriers to healthy eating among underserved populations and inspired this research.

**Methods:**

The effectiveness of spices and herbs in improving diet quality among underserved populations was evaluated in a series of studies conducted among samples of low-income and predominantly Black high school students in Baltimore, Maryland. A controlled study compared the effectiveness of school-based nutrition education focusing on spices and herbs (“Spice MyPlate” intervention) with conventional nutrition education. Diet quality was assessed via 3-day written food records. Another school-based study in Baltimore compared National School Lunch Program (NSLP) vegetable intake with and without the addition of spices and herbs. Monadic sensory testing was conducted to evaluate sensory preferences, and vegetable intake was assessed via plate waste. Inspired by the success of these studies, the research was extended to junior military service members, a population also known to have low diet quality, at Naval Support Activity Bethesda (NSAB). Monadic sensory testing was conducted to evaluate sensory preferences and vegetable intake in “heat and serve” meal kits was assessed via remote food photography methods.

**Results:**

The Spice MyPlate school-based nutrition education program was feasible, and there were improvements in the Spice MyPlate group compared with standard nutrition education in intake of whole grains and protein foods. The addition of spices and herbs to NSLP vegetable offerings resulted in an increase in total vegetable intake compared with otherwise identical vegetable preparations without spices and herbs. Vegetables with spices and herbs were preferred over otherwise identical preparations among military service members at NSAB.

**Discussion:**

Spices and herbs are promising for improving diet quality among a variety of underserved populations. The improvements may be mediated by the sensory enhancing properties of affordable and widely available spices and herbs.

## INTRODUCTION

The overall diet quality of the United States population has consistently fallen short of the recommendations provided in the Dietary Guidelines for Americans (DGA). The Healthy Eating Index (HEI) reveals concerning trends in American dietary patterns. Current data indicate the average HEI-2020 score for Americans is only 58 out of a possible 100 points.^1^ The diet quality of children and adolescents is particularly concerning because more than half of American children consume poor-quality diets.^2^

Underserved populations in the United States are further burdened by a complex interplay of socioeconomic, cultural, and environmental determinants that exacerbate challenges to diet quality. Individuals residing in low‐income communities, racial and ethnic minorities, and those living in urban and rural areas frequently contend with “food deserts,” areas with limited access to full‐service grocery stores that offer high‐quality, nutrient‐dense foods. They are concurrently often exposed to an abundance of fast-food outlets and corner stores selling predominantly energy‐dense, nutrient‐sparse options.^3^ Geographic and economic constraints interact such that communities of low socioeconomic status are more likely to experience food insecurity, which, in turn, is strongly associated with lower HEI scores.^4^ As is the case more broadly, young people in underserved communities are particularly burdened with poor diet quality.^5^

The goal of our research was to evaluate the effectiveness of spices and herbs in helping improve diet quality among underserved populations, with a focus on younger people. We hypothesized that the sensory-enhancing properties of spices and herbs could help overcome the common intake barriers of taste, aroma, and appearance of healthy foods.

Education is known to play a critical role in shaping dietary behaviors, and people less familiar with federal dietary guidance have lower HEI scores.^6^ Accordingly, this research began with a controlled, school-based nutrition education intervention focusing on spices and herbs (the “Spice MyPlate” intervention) that was conducted among a sample of low-income and predominantly Black high school students in Baltimore, Maryland. The research team then continued the school-based research in Baltimore with a comparison of National School Lunch Program (NSLP) vegetable intake with and without the addition of widely available and affordable spices and herbs. Inspired by the success of these studies in improving diet quality, we then aimed to replicate the NSLP research approach among a sample of junior military service members at Naval Support Activity Bethesda (NSAB). Young military service members are another underserved population shown to have poor diet quality,^7^ due in large part to living and working in fast-paced environments that prioritize the organizational mission over health, coupled with a preponderance of fast-food and nutrient-sparse “grab and go” meal options.^8^ In addition, there are financial barriers to fruit and vegetable consumption among junior military service members.^8^^,^^9^ Food insecurity disproportionally affects members of the US military compared with civilian-matched control individuals (25.3% vs 10.1%).^10^ Furthermore, enlisted service members with less than 4 years of service (junior enlisted) who lived on base were at the highest risk for food insecurity (30%).^11^ Food insecurity has been associated with poor dietary quality, specifically lower consumption of vegetables,^12^^,^^13^ which contributed to the motivation for extending this intervention approach to junior military service members.

This article appears as part of the supplement “The Role of Spices and Herbs on Supporting Healthy Diets and Improving Nutritional Status,” sponsored by the McCormick Science Institute.

## METHODS

The methods of each of the 3 studies in this research portfolio were approved by the University of Maryland School of Medicine and/or the Uniformed Services University of the Health Sciences institutional review boards.

### Spice MyPlate Nutrition Education Intervention

The Spice MyPlate nutrition education intervention is described in detail elsewhere.^14^ In brief, a high school–based intervention was conducted in the spring of 2013 comparing standard nutrition education according to the US Department of Agriculture (USDA) MyPlate guidelines (control arm) with standard nutrition education plus the Spice MyPlate intervention (intervention arm).

The study was conducted at 2 public high schools in Baltimore, Maryland. The 2 schools were similar with respect to socioeconomic status, racial minority composition, location, and the high prevalence of food deserts in the surrounding neighborhoods where students resided. All students in grades 9–12 from both participating high schools who could read and write in English were eligible to participate in this study. Informed written consent from parents or legal guardians and written assent from all participating students were obtained prior to the onset of the study.

The development and evaluation of the Spice MyPlate intervention was informed by extensive engagement with stakeholders from the participating schools and included students, parents and guardians, health educators, and school administrators. Participants in the control group received 1 hour of nutrition education on the USDA MyPlate guidelines offered during health education class. The nutrition education session was representative of the nutrition education offered in Baltimore high schools and comprised general instruction on USDA MyPlate, practical ways to eat in alignment with MyPlate, and how to record food intake using 3-day food records (the dietary assessment used in this study). Participants in the Spice MyPlate group received the standard nutrition education plus 6 weekly sessions during health education class that were 1 hour long and focused on using spices and herbs to eat a diet in alignment with MyPlate guidelines. Twelve widely available and relatively low-cost spices and herbs—cinnamon, black pepper, red pepper, basil, garlic, oregano, thyme, nutmeg, ginger, turmeric, rosemary, and cumin—were chosen as the focus of the Spice MyPlate curriculum. The education was highly experiential and included creation of recipes and flavor combinations with spices and herbs, cooking sessions with a professional chef focused on using spices and herbs, and group activities.

Dietary intake was assessed using 3-day food records including 2 weekdays and 1 weekend day. Participants in both study arms provided completed food records before attending the standard nutrition education session received by both arms, and at 3, 4, and 6 weeks after completing the intervention. A registered dietitian used the USDA SuperTracker tool^15^ (no longer available) to quantify food record data into amounts of each of the 5 components of MyPlate.

The primary outcomes in this study were the intake of each of the metrics of diet quality in alignment with the Dietary Guidelines for Americans and MyPlate: vegetables (cups), fruits (cups), whole grains (ounces), dairy (cups), and protein foods (ounces). Secondary outcomes were participants’ attitudes toward eating each of the components of MyPlate with and without spices and herbs, assessed by Likert-scale questionnaires provided to and self-reported by participating students.

### NSLP Spices, Herbs, and Vegetables Intervention

The NSLP spices, herbs, and vegetables intervention is described in depth elsewhere.^16^^,^^17^ In brief, a 2-phase intervention was conducted at a high school in Baltimore during the 2015–2016 and 2016–2017 academic years.

Phase 1 took place in 2015–2016 and consisted of stakeholder engagement and monadic sensory-testing of vegetable preparations with and without spices and herbs. The stakeholder engagement process began with meetings between the research team, school administration, and food services personnel to discuss the feasibility of potential intervention approaches aimed at increasing vegetable intake among students. This process was then extended to students, parents, and guardians.

Students were then invited by school staff to participate in an after-school program that included education on sensory effects, exposure to spices and herbs, sensory enhancement of vegetables with spices and herbs, and sensory testing of vegetable recipes prepared with spices and herbs. The program consisted of 10 sessions that were each 1.5 hours long. Nutrition and sensory education was provided at each session and 2 to 4 vegetable recipes were sensory tested and rated for flavor, appearance, odor, and texture. The sensory testing compared “typical” vegetable recipes (only NSLP-compliant amounts of oil and salt typical of what was served in the school cafeteria) to otherwise identical “spiced” recipes (same amounts of oil and salt plus spices and herbs). Recipes were rated on a 1 to 5 Likert scale for each sensory domain and an overall preference was provided by the students. The recipes prepared with spices and herbs that received the highest mean ratings were selected for the vegetable intake comparison in phase 2 vs the typical vegetable recipes. The spices and herbs that most consistently appeared in the sensory-tested spiced vegetable intervention recipes included onion powder, garlic powder, dill weed, black pepper, and cayenne pepper.

Phase 2 took place in 2016–2017 and consisted of a school cafeteria-based comparison of NSLP vegetable intake with and without spices and herbs. As in phase 1, the typical vegetables were steamed and included oil and salt in alignment with NSLP guidelines. The spiced recipes were prepared in an identical manner to the typical vegetables and included the same amounts of oil and salt and the addition of spices and herbs. The identical preparation methods and oil and salt content were used to isolate the inference to the addition of spices and herbs as closely as possible. The vegetables assessed in the intake comparison were broccoli, steamed carrots, raw carrots, California medley (broccoli, carrots, cauliflower), black beans and corn, peas, and green beans.

Each student was provided a serving of vegetables at lunch every day. Vegetables were served in a separate container on each student’s lunch tray to allow for precise estimation of the vegetables the student did not consume. Separate vegetable containers obviated the need for research staff to manually separate vegetables from other foods served at lunch. Students returned their lunch trays to research staff at trash-can stations in the cafeteria to ensure collection of all student lunch trays. Research staff separated and delivered the vegetable containers to other research staff who weighed the vegetables. Weighed vegetable plate waste was selected for the primary outcome of the study because it has been used in many previous cafeteria studies. Two OHAUS Gold Series SPJ601 scales (OHAUS, Parsippany, NJ) were used by study staff to weigh the vegetable plate waste. The daily served weight for each vegetable was also estimated by calculating the mean weight of 10 separate servings provided by cafeteria staff to the research team on each day of the intervention.

Vegetable intake was assessed in this way during 2 4-week periods in November–December (fall) and April–May (spring). The typical vegetables were served and collected for weighing for 2 consecutive weeks during each 4-week period, followed by the spiced vegetables being served and collected for weighing for another 2 consecutive weeks. These data collection schedules resulted in approximately 40 lunch periods in which student vegetable intake was assessed in the fall and another 40 lunch periods in which student vegetable intake was assessed in the spring. Mean vegetable intake (in grams) was calculated as the difference between the estimated serving weight and the vegetable plate waste weight returned on each student’s tray. Willingness to try each served vegetable was also assessed as either yes (lower vegetable plate waste weight than estimated serving weight) or no (equal or higher vegetable plate waste weight than the estimated serving weight).

### Military Spices, Herbs, and Vegetables Intervention

The military spices, herbs, and vegetable intervention is described in greater detail elsewhere.^18^ In brief, the research approach of the NSLP intervention in Baltimore was replicated in a 2-phase intervention conducted at NSAB from 2021 to 2024. NSAB is a military base located in Bethesda, Maryland, that includes Walter Reed National Military Medical Center, the Uniformed Services University, and the Warrior Transition Brigade, and supports more than 40 tenant commands.

Phase 1 was conducted in 2021 and 2022 and consisted of stakeholder engagement and monadic sensory testing of vegetable preparations with and without spices and herbs. The stakeholder engagement process involved meetings between the research team and military service members, the health promotion specialist on base, a military culinary specialist, staff dietitians, base senior leaders, and the base commander. These meetings helped inform intervention implementation and recruiting efforts, and provided clarity on the vegetable recipes currently being served in military dining facilities.

It was determined that the sensory testing would best be conducted at the United Service Organization, a popular and centrally located gathering space at NSAB. Participants first completed a series of questionnaires assessing modifiable barriers to consuming military dining facility vegetables and rating characteristics of the military vegetable preparations on a 5-point Likert scale. This assessment was followed by the sensory testing, which featured spiced vegetable recipes prepared in the same manner and containing the same ingredients as the typical vegetable recipes, with the exception of the addition of spices and herbs. Broccoli, carrots, cauliflower, and kale were used in the testing. The spiced vegetables tested included savory broccoli (garlic powder, onion powder, black pepper, and mustard seed), curry cauliflower (garlic powder, onion powder, turmeric, coriander), sweet spice carrots (vanilla and cinnamon), and smoky kale (garlic powder, onion powder, smoked paprika, and oregano). All vegetables in the sensory testing were initially steamed and then frozen along with a frozen compound butter containing butter and salt (typical) or butter and salt with spices (spiced). The frozen vegetables were microwaved, which melted the compound butter into the vegetables and allowed for even distribution of spices and herbs. Serving order was randomized to minimize expectation bias. Participants were also provided water and table crackers in between vegetables samples to cleanse their palate.

The United States Military—Menu Item Survey was used to evaluate the typical and spiced vegetable recipes in the sensory testing process. This survey contains a 9-item hedonic scale rating of appearance, aroma, flavor, texture, and overall appeal and is used by the Combat Feeding Division, Armed Forces Recipe Services when developing and evaluating new recipes to be used in military dining facilities. Paired t tests were used to compare overall liking and specific sensory characteristics between the vegetables with and without spices and herbs.

Phase 2 was conducted in 2023 and 2024 and featured a comparison of vegetable intake between typical and the spiced recipes evaluated in phase 1. To offer as much potential generalizability to real-world military eating behaviors as possible, popular “heat and serve” meal kits were provided to study participants for intake evaluation. The meal kits contained 2 cups of vegetables, either prepared with spices and herbs (“spiced”) or without spices and herbs (“typical”), alongside a consistent starch (0.5 cups of mashed potatoes) and protein (5-ounce grilled chicken breast). The consistency of the other meal components minimized the potential for confounding of vegetable intake by differing accompanying meal components. The same vegetable sensory profiles (savory broccoli, sweet spice carrots, curry cauliflower, and smoky kale) and preparation styles (frozen with compound butter) were used as in phase 1.

Fifty frozen “heat and serve” meal kits, featuring either the spiced or typical preparations of 1 of the 4 vegetables, were distributed at each of 9 intervention sessions. Although blinding was not possible, the vegetables were not titled or labeled in any manner to avoid drawing attention to any differences in spice content. Participating service members were not required to consume the meals on premises, to preserve the typical military dining setting as closely as possible. The vegetable preparation style (typical or spiced) offered at each session was determined through randomization. Each meal kit included a quick response code that brought participants to a SurveyMonkey webpage, where they anonymously uploaded photos of their completed meals after consumption and rated their liking of the vegetables using a 9-point Likert scale. SmartIntake Technology (Pennington Biomedical Research Center, Louisiana State University, Baton Rouge, LA), a validated form of the remote food photography method,^19^ was used to assess vegetable intake.

### Statistical Analyses

Unadjusted differences in intake of each of the 5 food groups in MyPlate (vegetables, fruits, whole grains, dairy, and protein foods) between the Spice MyPlate and control groups were estimated using *t* tests. Multivariate regression modeling with generalized estimating equations (GEEs) was also performed to account for potential confounding of within-subject intake correlation over time. In brief, GEE models were run for weekly intake of each of the 5 food groups of MyPlate; the variables included in each model were chosen a priori and included study group allocation, study visit, baseline intake of the food group being modeled, race, age, and gender. Within-subject correlation was accounted for in SAS software using the PROC GEE SUBJECT correlation structure. Differences in changes in the attitudes toward healthy eating were estimated using Wilcoxon-Mann-Whitney tests.

In the study involving high school students, comparison of intake between the typical and spiced vegetables was performed using Student’s *t* test. Comparison of willingness to try the typical and spiced vegetables was conducted using a χ^2^ test. All statistical analyses were performed individually for each semester and then assessed for heterogeneity via a generalized linear regression model with a product term. Regression models of intake and willingness to try vegetables, including semester by spiced interaction terms, were also constructed. In the study including military service members, comparisons of mean vegetable liking and intake between typical and spiced vegetable preparations were performed using *t* tests.

## RESULTS

### Spice MyPlate Nutrition Education Intervention

A total of 110 participants enrolled in the study of high school students, with 55 participants in both the Spice MyPlate and control arms. The sample was predominantly Black students (75%) with a relatively even distribution of male and female participants. Baseline diet quality and attitudes toward healthy eating were both relatively low in the Spice MyPlate and control arms. With the exception of whole-grains intake being slightly higher in the control arm, there were few differences in diet quality or attitudes toward health eating between study arms at baseline.

Participants in the Spice MyPlate arm reported a greater difference than the control arm in the intake of whole grains (31.2 g/week; *P* = .02) and protein foods (13.2 ounces/week; *P* = .004) at the 10-week follow-up, even after adjustment for covariates. There were no differences in the intake of vegetables, fruit, or dairy reported between the Spice MyPlate and control arms (*P* > .06).

Participants in the Spice MyPlate arm reported being more likely to eat vegetables (*P* = .03), lean protein (*P* = .006), and low-fat dairy (*P* = .01) at follow-up than at baseline. There were no changes reported in the control arm (*P* > .4). Participants in the Spice MyPlate group also reported they would be more likely to eat vegetables (*P* = .01) and whole grains (*P* = .05) at follow-up than at baseline. Again, there were no changes reported in the control arm (*P* > .2).

### NSLP Spices, Herbs, and Vegetables Intervention

A total of 26 high school students participated in the stakeholder engagement and sensory testing of phase I of this study. The sample was predominantly Black students (92%) and there were more female (62%) than male participants. Questionnaires revealed that preparation method (68%) and taste (55%) were common barriers for not eating NSLP vegetables. The participants suggested that modifying the flavor (44%), appearance (12%) and preparation method (12%) would be helpful to increase NSLP vegetable consumption. Sensory testing revealed that spiced versions of all vegetable preparations under study (black beans and corn, broccoli, California blend [broccoli, cauliflower, carrots], and peas) were preferred over the typical preparations (*P* < .05), with the exception of carrots, for which the spiced and typical versions were rated exactly equally.

A total of 4570 plates were collected and included for plate waste analysis in the cafeteria-based vegetable intake comparison of phase 2 of this study. Overall, vegetable intake was 18.2% higher with spices and herbs than typical preparations (1.58 ounces of typical vegetables; 1.87 ounces of vegetables prepared with spices and herbs; *P* < .0001). Intake of 5 of the vegetables tested was higher with spices and herbs (broccoli, California medley, green beans, raw carrots, steamed carrots) and 2 vegetables tested had higher intake with typical recipes (black beans and corn, peas). There were preparation inconsistencies in the school cafeteria with the recipes for peas (spices and herbs were not evenly distributed) and black beans and corn (recipe was not scaled properly from sensory-tested version in phase 1, and there was an excessive amount of liquid when served to students) that were believed to have confounded the intake comparison. The analysis of vegetable intake limited to recipes in which preparation was consistent (broccoli, California medley, green beans, raw carrots, steamed carrots) revealed greater increases in vegetable intake with spices and herbs as compared with typical recipes across all vegetables under study (34.2%; 0.53 ounce; *P* < .0001).

### Military Spices, Herbs, and Vegetables Intervention

A total of 70 active-duty military service members participated in the stakeholder engagement and vegetable sensory testing of phase 1 of this study. The mean age of the study participants was 27.4 years, and the sample was diverse with respect to race/ethnicity (37% White, 19% Hispanic, 17% Black, 15% Asian, 12% mixed/other race/ethnicity), sex (54% male, 46% female), and rank (62% officer, 38% enlisted). The most common barriers to vegetable intake in the military dining facility, as reported in questionnaires, were appearance (42.9%), preparation style (41.3%), and taste (39.7%). Across all vegetables under study, sensory testing revealed that spiced vegetables were preferred to the typical preparations in overall appeal, flavor, and aroma (*P* < .03). The overall appeal reported for the spiced broccoli, carrots, and cauliflower was greater than for the typical vegetables (*P* < .0002). There was no difference in overall appeal between the spiced and typical kale (*P* = .3). Aroma was the sensory domain that was most positively impacted by the addition of spices and herbs. The aroma of the spiced vegetables was preferred to that of the typical preparations for all vegetables under study (*P* < .03). The flavor and appearance of spiced vegetables were also preferred over the typical preparations for most vegetables tested. Texture was the sensory domain least affected by the addition of spices and herbs. The typical preparations were not preferred in any domain for any vegetable in comparison to the spiced vegetables.

The data from the vegetable intake analysis are currently under review and are not presented here. In brief, the heat-and-serve meal kits offered and measured in this intervention featured encouragingly high levels of vegetable intake with both the typical and spiced preparations, reflecting the popularity of this vehicle to overcome barriers to vegetable intake by the service members.

## DISCUSSION

Seasoning with spices and herbs appears to be feasible and effective for improving diet quality among underserved populations that frequently struggle with a variety of barriers to healthy eating, spanning financial constraints, lack of local access to healthy food, limited nutrition KSAs, and other challenges. To foster the potential continuity of the interventions after the completion of the studies and replicability in other settings, each study explicitly focused upon spices and herbs that are widely available and relatively affordable for the individuals and institutions under study.

We think there are several contributing factors underlying the improvements in diet quality and attitudes toward healthy eating noted in this series of studies. The sensory-enhancing properties of spices and herbs are likely a primary driver of these improvements. Among the most potent and consistent barriers to vegetable intake reported across the different populations in these studies were the unappealing taste, appearance, and aroma of vegetables and other healthy foods. Our results suggest these barriers to healthy eating were present in the home but were most prominent among the institutional food offerings (eg, school lunch, military base dining options). An extensive body of evidence that has established the ability of spices and herbs to enhance these sensory properties of healthy foods in other populations^20^^,^^21^ supports this hypothesized mechanism.

Another factor contributing to the improvements in diet quality and attitudes toward healthy eating, particularly among the high school populations studied, is the versatile opportunity for personal expression in healthy eating offered by the addition of spices and herbs. Students in the Spice MyPlate study, in particular, enjoyed the opportunity to use common spices and herbs to create their own unique flavor profiles in healthy foods they prepared both at school and at home. This sense of ownership in the process and opportunity for personal expression in healthy eating was noted to increase student interest in what, heretofore, was considered to be a relatively dull subject.

Although we think spices and herbs may be equally promising vehicles for improving diet quality in other underserved populations, and numerous efforts were implemented to minimize nutritional (eg, preparation style, oil and salt content) and contextual (eg, labeling, promotion of benefits of spices) sources of confounding, blinding was a limitation in the sensory and preference testing in these studies. However, the appearance, aroma, and flavor of spices and herbs that prevent blinding are themselves the key features that help improve sensory profiles of vegetables that support intake in challenging populations. A key to the feasibility of these studies was the engagement with key stakeholders in each of the study settings prior to intervention. The stakeholders in each study setting identified unique barriers to diet quality and appreciated the opportunity to express their opinions with respect to how the research team might best implement the intervention and achieve lasting success. It should be noted that both urban schools and military bases have a long history of challenges with diet quality. We were cognizant of the fact that the experiences and insights offered by the stakeholders would increase the likelihood of successful implementation and outcomes. It is likely that some adaptation of the intervention approaches described in these studies will be helpful in achieving success in other underserved settings.

Such studies are needed to replicate these findings in other underserved settings, but this series of studies suggests that both nutrition education and direct intervention adding common spices and herbs to healthy foods appear to be feasible and effective interventional strategies for improving diet quality and attitudes toward healthy eating among underserved populations.
